# SplicingLore: a web resource for studying the regulation of cassette exons by human splicing factors

**DOI:** 10.1093/database/baad091

**Published:** 2023-12-21

**Authors:** Hélène Polvèche, Jessica Valat, Nicolas Fontrodona, Audrey Lapendry, Valentine Clerc, Stéphane Janczarski, Franck Mortreux, Didier Auboeuf, Cyril F Bourgeois

**Affiliations:** Laboratoire de Biologie et Modelisation de la Cellule, Ecole Normale Superieure de Lyon, CNRS, UMR 5239, Inserm, U1293, Universite Claude Bernard Lyon 1, 46 allee d’Italie, Lyon F-69364, France; Equipe Labellisee Ligue Contre le Cancer, 4 allee d'Italie, Lyon 69007, France; Laboratoire de Biologie et Modelisation de la Cellule, Ecole Normale Superieure de Lyon, CNRS, UMR 5239, Inserm, U1293, Universite Claude Bernard Lyon 1, 46 allee d’Italie, Lyon F-69364, France; Equipe Labellisee Ligue Contre le Cancer, 4 allee d'Italie, Lyon 69007, France; Laboratoire de Biologie et Modelisation de la Cellule, Ecole Normale Superieure de Lyon, CNRS, UMR 5239, Inserm, U1293, Universite Claude Bernard Lyon 1, 46 allee d’Italie, Lyon F-69364, France; Equipe Labellisee Ligue Contre le Cancer, 4 allee d'Italie, Lyon 69007, France; Laboratoire de Biologie et Modelisation de la Cellule, Ecole Normale Superieure de Lyon, CNRS, UMR 5239, Inserm, U1293, Universite Claude Bernard Lyon 1, 46 allee d’Italie, Lyon F-69364, France; Equipe Labellisee Ligue Contre le Cancer, 4 allee d'Italie, Lyon 69007, France; Laboratoire de Biologie et Modelisation de la Cellule, Ecole Normale Superieure de Lyon, CNRS, UMR 5239, Inserm, U1293, Universite Claude Bernard Lyon 1, 46 allee d’Italie, Lyon F-69364, France; Laboratoire de Biologie et Modelisation de la Cellule, Ecole Normale Superieure de Lyon, CNRS, UMR 5239, Inserm, U1293, Universite Claude Bernard Lyon 1, 46 allee d’Italie, Lyon F-69364, France; Equipe Labellisee Ligue Contre le Cancer, 4 allee d'Italie, Lyon 69007, France; Laboratoire de Biologie et Modelisation de la Cellule, Ecole Normale Superieure de Lyon, CNRS, UMR 5239, Inserm, U1293, Universite Claude Bernard Lyon 1, 46 allee d’Italie, Lyon F-69364, France; Equipe Labellisee Ligue Contre le Cancer, 4 allee d'Italie, Lyon 69007, France; Laboratoire de Biologie et Modelisation de la Cellule, Ecole Normale Superieure de Lyon, CNRS, UMR 5239, Inserm, U1293, Universite Claude Bernard Lyon 1, 46 allee d’Italie, Lyon F-69364, France; Equipe Labellisee Ligue Contre le Cancer, 4 allee d'Italie, Lyon 69007, France; CECS/AFM, I-STEM, 28 rue Henri Desbrueres, Corbeil-Essonnes F-91100, France

## Abstract

One challenge faced by scientists from the alternative RNA splicing field is to decode the cooperative or antagonistic effects of splicing factors (SFs) to understand and eventually predict splicing outcomes on a genome-wide scale. In this manuscript, we introduce SplicingLore, an open-access database and web resource that help to fill this gap in a straightforward manner. The database contains a collection of RNA-sequencing-derived lists of alternative exons regulated by a total of 75 different SFs. All datasets were processed in a standardized manner, ensuring valid comparisons and correlation analyses. The user can easily retrieve a factor-specific set of differentially included exons from the database or provide a list of exons and search which SF(s) control(s) their inclusion. Our simple workflow is fast and easy to run, and it ensures a reliable calculation of correlation scores between the tested datasets. As a proof of concept, we predicted and experimentally validated a novel functional cooperation between the RNA helicases DDX17 and DDX5 and the heterogeneous nuclear ribonucleoprotein C (HNRNPC) protein. SplicingLore is available at https://splicinglore.ens-lyon.fr/.

**Database URL:**  https://splicinglore.ens-lyon.fr/

## Introduction

Eukaryotic genes are transcribed into pre-mRNAs that are most of the time composed of a succession of exons separated by introns, the latter being removed by the spliceosome to form mature RNA molecules or mRNAs. The spliceosome is a large ribonucleoprotein complex, which recognizes the 5′ and 3′ splice sites delimitating exons from introns and catalyzes the splicing reaction, a process that is modulated by numerous auxiliary factors, including RNA binding proteins (RBPs) (1, 2). The split nature of genes and the contribution of many different parameters enable alternative splicing, which is defined as the differential selection of exonic or intronic sequences to produce different mRNA isoforms from the same gene. Alternative splicing is a prevalent phenomenon that greatly expands proteome diversity and also contributes to the quantitative regulation of gene expression (3, 4). Various splicing-dedicated databases, such as VastDB (5), ASpedia (6) or FasterDB (7), serve as repositories of alternative splicing profiles and inclusion levels across cell types or organisms. ASpedia also provides useful functional information about the alternative exons, as Exon Ontology (8) that helps to predict the biological consequences of genome-wide splicing variations.

Mechanistically, the binding of RBPs, like serine/arginine-rich (SR) proteins and hnRNP proteins, to short degenerated RNA motifs located within exons or in their flanking introns, is determinant for the control of alternative exon inclusion (9–12). The activity of a given RBP can also be favoured or antagonized by other factors, hereafter defined generally as splicing factors (SFs), and their joint action or competition can promote or inhibit the assembly of the spliceosome onto nearby splice sites (13, 14). The specificity and intrinsic properties of each SF, even those of general constituents of the spliceosome, combined to the unique sequence and structural organization of each exon, explain why the knockdown of a specific factor in cells only results in the differential splicing of a limited number or transcripts, highlighting the relative plasticity of the spliceosome. The identification and quantification of splicing changes controlled by a given factor can be monitored by RNA sequencing (RNA-seq) technologies (15). However, it is more difficult to understand or to predict the cooperative or antagonistic effects of SFs on alternative splicing, especially on a genome-wide scale.

To address this question, we present SplicingLore, a database and web resource that contain the lists of alternative exons that are differentially included upon knockdown of 75 different SFs in various human cell lines. This collection is integrated into a website from which the user can easily download a given dataset of SF-regulated exons or submit a single exon or a list of exons to predict their potential regulation by the SFs of the database. As a proof of concept of the utility of our resource, we predicted and experimentally validated a new functional cooperation between RNA helicases DDX17 and DDX5 and the HNRNPC protein. SplicingLore is freely available at https://splicinglore.ens-lyon.fr/.

## Results

### Data collection and analysis

**Figure 1.**
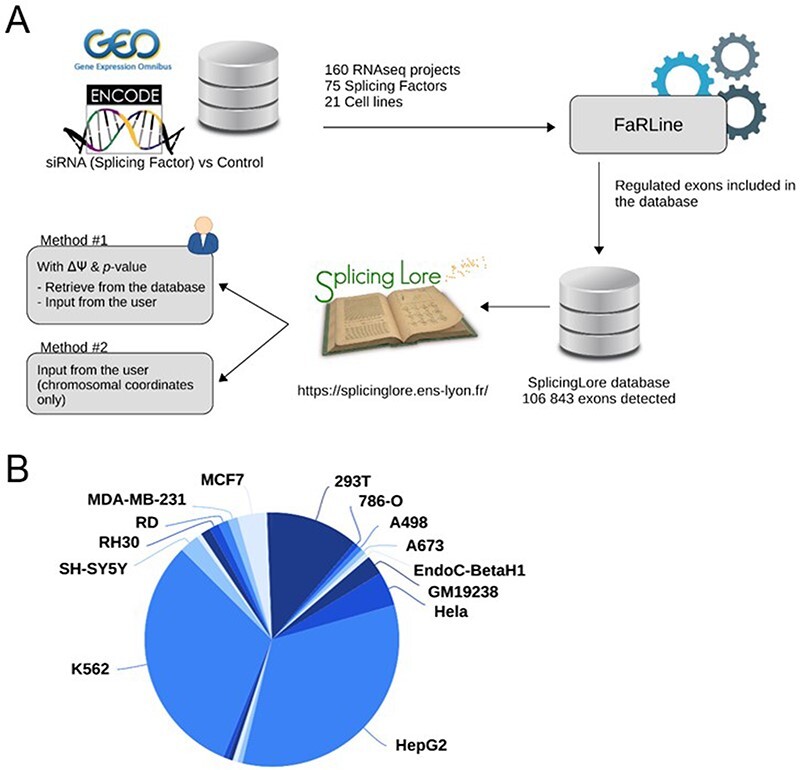
General features of SplicingLore. (**A**) Outline of the SplicingLore workflow. RNA-seq datasets from GEO and ENCODE databases were uniformly processed with FaRLine to generate lists of alternative exons regulated by 75 SFs in 21 human cell lines. These datasets can be used to compute correlation scores of exon regulation with a list of exons of interest, which is either retrieved from the database or provided by the user (Query Method 1). The user can alternatively query SplicingLore with a list of exons associated only with their chromosomal coordinates (Method 2). In the latter case, the list of exons can include or not a ΔPSI and *P*-value. (**B**) Diagram showing the repartition of the SplicingLore datasets between cell lines.

To feed the SplingLore database, we searched the GEO omnibus (16) and ENCODE (17) databases for datasets generated from Illumina RNA-seq experiments that all consisted in knocking down a given SF in a human cell line, mostly by means of a treament with small interfering RNA (siRNA) or short hairpin RNA (shRNA) (Figure 1A). We filtered out the datasets for which the sequencing quality or depth was too low to reach our objective, which was to obtain robust and reliable lists of alternative exons regulated by the tested SF (see Methods for details). We eventually obtained a curated list of 160 datasets, corresponding to 75 SFs tested in 21 different cell lines (Figure 1A, Supplementary Table S1). Three cell lines (HepG2, K562 and 293T) represented more than 75% of the datasets (Figure 1B), and some SFs were tested in up to five cell lines.

**Alt text for **  Figure 1: Description of the SplicingLore workflow and different methods of analysis, featuring the repartition of the datasets between different cell lines.

The selected datasets were all processed in the same way using FaRLine, a splicing-dedicated pipeline developed earlier in our lab (18). We thus obtained uniformly formatted lists of alternative splicing events for each dataset. Although FaRLine detects and quantifies variations in alternative 5′ and 3′ splice sites, as well as multiply spliced exons, we decided to focus only on single-cassette exons, which represent the vast majority of regulated splicing events and are easier to handle when comparing multiple datasets. Note that the annotation of all exons is based on the FasterDB database (https://fasterdb.ens-lyon.fr/faster/home.pl) (7).

Integrating data from various cell lines could introduce a bias as splicing regulation is inherently variable across cell lines. Indeed, some exons may exhibit a discordant regulation by the same SF in two different cell lines, most likely because cofactors may contribute to their cell-specific regulation. To address this question, we looked whether differential exon inclusion (DeltaPSI; PSI: percent spliced-in) changed across cell lines, for each SF analysed in at least three datasets. These analyses globally showed that each SF displayed a similar regulation of its target exons across different cell lines (Supplementary Figure S1). We then performed a broader clustering analysis for all exons regulated by all SFs for which at least three cell lines were available. This analysis showed that exons were clustered according to the SF rather than the cell line (Supplementary Figure S2), ruling out a possible cell line-based bias. Note that the analysis presented in Supplementary Figure 1 also allowed us to verify that the way the cells were treated to achieve SF knockdown (mostly siRNA or shRNA) did not significantly affect the result.

**Figure 2.**
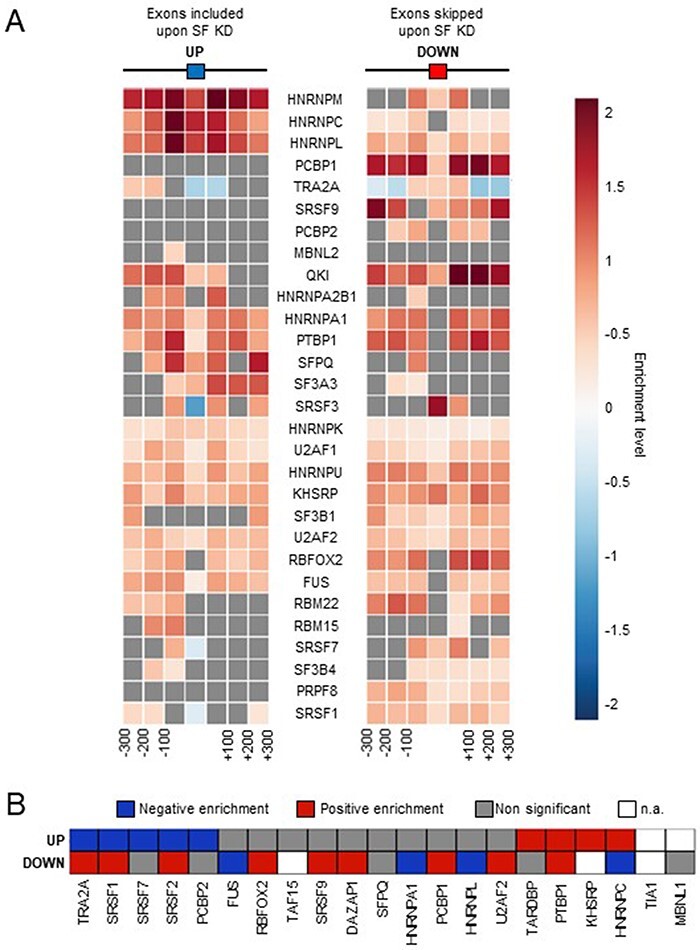
Comparison with SF binding sites. (**A**) Enrichment of SF binding sites (based on CLIP-seq data) at exons that are up- or down-regulated by the corresponding factor and in their flanking regions. Each square on both sides of the exon represents a 100 nucleotide window upstream and upstream of the exon. (**B**) Enrichment of SF-specific binding sequences at exons (±200 nucleotides) that are up- or down-regulated by the corresponding factor. Not applicable (n.a.): less than 100 exons were identified by FaRLine as more included or excluded upon knockdown of the corresponding SF.

To underline further the relevance of our alternative splicing analyses, we also tested whether SF binding was enriched in the regions surrounding their corresponding alternative exons identified by FaRLine. First, we recovered available cross-linking and immunoprecipitation (CLIP)-seq data for 29 SFs and calculated the enrichment of peaks at and around the exons that are up- and down-regulated by the corresponding factors, as determined by FaRLine, compared to control exons. As shown in Figure 2A, exons regulated by each tested SF displayed an enriched binding of the corresponding SF at an/or around the exon, with sometimes a differential enrichment pattern between positively and negatively regulated exons. For example, HNRNPC binding is strongly enriched at exons upregulated upon HNRNPC KD, consistent with its general binding-associated repressive role on splicing (12, 19). In contrast, SR proteins such as SRSF3, SRSF1, SRSF7 or TRA2A bind more strongly to the exons that they promote the inclusion of (Figure 2A) (20). Second, we recovered the consensus binding sites for 21 SFs and similarly looked for their enrichment at/around their down- and up-regulated exons (Figure 2B). Again, the differential enrichment of binding sites matched the known function of the corresponding SF. For example, we observed a positive enrichment of SR protein binding sites on exons that are positively regulated by these proteins and vice versa.

**Alt text for **  Figure 2: Heatmaps showing the relative enrichment of SF binding sites or binding motifs around exons that are up- or down-regulated by the same factor.

### Web interface and graphic visualization

**Figure 3.**
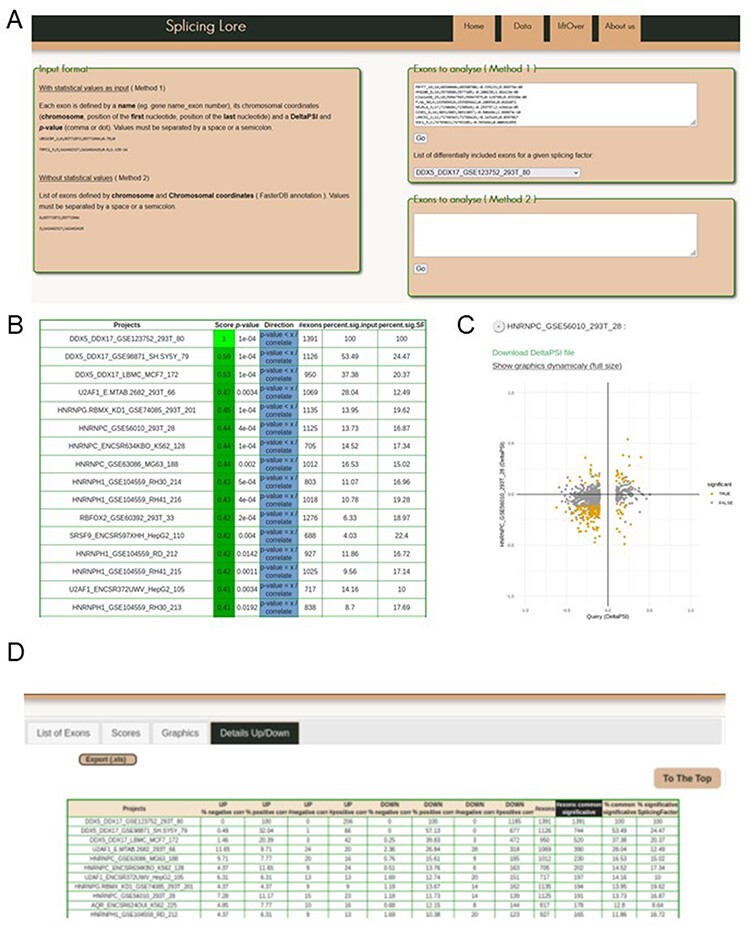
Visualization of the SplicingLore web interface. (**A**) View of the home page, with the instructions to prepare the input list of exons and the query window. This includes the drop-down menu that allows to retrieve a given list of SF-regulated exons from the database. (**B**) View of the top of the main output file, showing for each dataset the different correlation parameters with the query list (here the exons regulated by DDX5/DDX17 in 293T cells). The Score and associated *P*-value for positive or negative correlation are provided for each dataset of the database, along with the total number of exons in the corresponding dataset and the fraction of common exons between the compared datasets, relative to the query list or to the tested dataset. (**C**) The correlation graph of the ∆PSI values for common exons from the query list and one of the stored datasets. Coloured dots represent exons that are significantly regulated by both SF. The file containing the corresponding ∆PSI values for these exons can be downloaded here. (**D**) View of the top of the detailed output file, which provides the number (#) and fraction (%) of shared exons between the query and the dataset, separated in up-regulated and down-regulated subclasses of exons.

SplicingLore can be queried in two different ways (Figures 1A and 3A). The first method allows the user to analyse a list of exons associated with parameters of splicing regulation. The default format requested for this analysis includes the gene symbol and exon number, chromosomal coordinates, as well as values of differential exon inclusion (ΔPSI) and *P*-value (Method 1, Figure 3A, left window). Gene symbols and exon numbers correspond to gene annotations of the FasterDB database (7), but the critical details to provide here are the chromosomal coordinates. Incorrect gene symbol and/or exon number will not impede the search but instead will issue a warning message and a proposition for correcting the input, based on coordinates (see later). To facilitate the navigation and the conversion of exon coordinates in both directions between the hg19 and hg38 version of the human genome, the SplicingLore interface includes a tab that allows the user to directly launch a conversion from the UCSC reference chain files with the R package liftOver (https://bioconductor.org/packages/liftOver) (Figure 3A).

**Alt text for **  Figure 3: Screenshots of the SplicingLore web interface and output files.

The query list of exons can be automatically retrieved from one of the projects referenced in the database, upon selection of the dataset of interest from the drop-down menu (Figure 3A, top right window). Alternatively, the user can upload a list of exons of interest to query SplicingLore for their potential regulation by the SFs of the database. As each exon is associated to a ΔPSI value, this first method will allow to look for positive and negative correlations between the input regulation and the regulation by SFs from the database.

The second query method is less restrictive as it does not require any parameter of exon inclusion (ΔPSI and *P*-value), if these values are not available (Method 2, Figure 3A). In this mode, the user can upload a list of exons that are only identified by their chromosomal coordinates (Figure 3A, bottom right window).

Once the query is launched, the first displayed page (‘List of exons’ tab) informs the user about exons that were not properly recognized, either because they were not detected in any of the 160 datasets or because they were incorrectly formatted. In the latter case, corrected features are given to allow the user to restart the analysis (Supplementary Figure S3).

From the ‘Scores’ tab, the user obtains a downloadable table (Figure 3B), which recapitulates the correlation scores between the query list and each of the SplicingLore datasets, as well as different comparison parameters that are explained later. Since this table is sorted based on our custom Score, the user can immediately visualize on top of the list which SF is more susceptible to be involved in the regulation of the tested exons. Scores indicating a significant positive or negative correlation are shown in different colours to facilitate their rapid visualization on the website (downloaded files are colourless). The ‘percent.common.query’ and ‘percent.common.SF’ columns indicate the fraction of common exons between the compared datasets, calculated from the query list or from the tested dataset, respectively (see Supplementary Figure S4 for a representation of these fractions).

The ‘Graphics’ tab presents the same information in the form of a correlation graph, which is available in a fixed or interactive mode (Figure 3C). The values used to generate these graphs can also be downloaded as a table. Finally, a more comprehensive table is available in the ‘Details Up/Down’ tab (Figure 3D). Alongside the information described earlier, it also provides the number (#) and fraction (%) of exons that are shared by both compared datasets, taking into account the direction of their regulation (up-regulation or down-regulation of their inclusion upon SF knockdown).

### Example of application

In order to check out the validity of our tool, we retrieved from SplicingLore the list of exons whose inclusion was altered upon the knockdown of DEAD-box RNA helicases DDX5 and DDX17 in the 293T cell line (GSE123752, Supplementary Table S3). We then used this list as a query to search for possible correlative (or anti-correlative) effects between these splicing regulators and other SFs.

The first lines of the resulting table show that beside the query list itself (score of 1), the best correlation scores correspond to the other two DDX5/DDX17 datasets included in SplicingLore, in SH-SY5Y and MCF7 cell lines, with respective scores of 0.59 and 0.53 (Figure 3B, Supplementary Table S4). Interestingly, we also observed a good correlation (with a score of 0.41–0.43) between the tested DDX5/DDX17 dataset and five different HNRNPH1 datasets (Figure 3B, Supplementary Table S4). This is in agreement with the fact that HNRNPH1 was previously described as a co-regulator of DDX5/DDX17-mediated splicing in myoblasts and epithelial cells (21).

Results from Supplementary Table S4 also revealed HNRNPC as one of the top predicted factors, with positive correlation scores in four datasets (score = 0.44 in 293T, K562 and MG63 cell lines and 0.38 in HepG2 cells) (Figure 3B, Supplementary Table S4). When considering separately the subclasses of up- and down-regulated exons, we noticed that the positive correlation between DDX5/DDX17 and HNRNPC exons was especially visible for down-regulated exons (Supplementary Table S5, Supplementary Figure S5). Indeed, up to 15.6% of exons down-regulated upon HNRNPC knockdown were regulated in the same manner (11.5% in average for the four correlated datasets), while only about 1% were regulated in the opposite manner (Supplementary Tables S5 and S6). For up-regulated exons, results were more variable between the different datasets, although a trend for a positive correlation was also observed, especially in 293T and K562 cells. These two datasets displayed more than 11% of positive correlation with the query, these values being the highest among all datasets (apart from DDX5/DDX17 datasets, Supplementary Table S5).

To explore further the possibility of a functional link between DDX5/DDX17 and HNRNPC, we first queried SplicingLore in a reverse manner, uploading on the website a list of 3030 exons corresponding to the union of all exons regulated upon HNRNPC knockdown in four datasets of our database (293T, K562, HepG2 and MG63). This analysis identified the three DDX5/DDX17 datasets among those showing a significant positive correlation (scores from 0.46 to 0.50) with the query list (Supplementary Table S7, Supplementary Figure S6).

**Figure 4.**
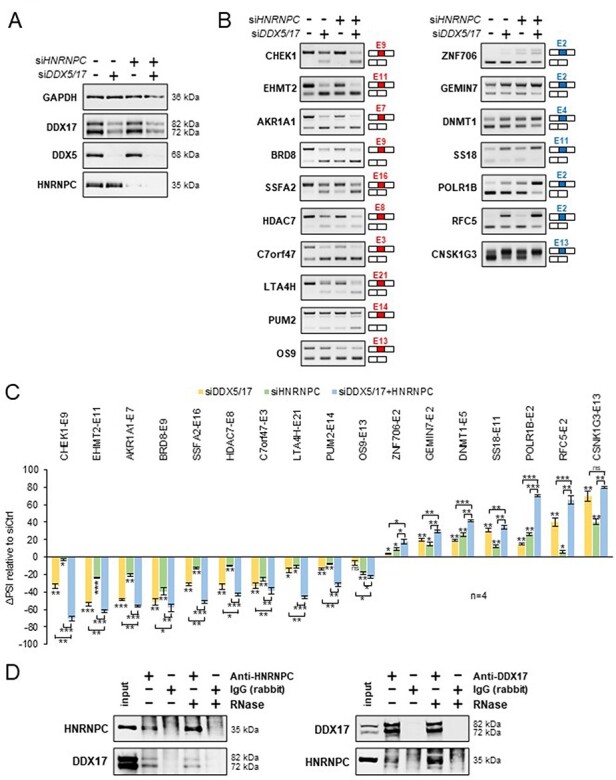
Validation of a functional relationship between DDX5/DDX17 and HNRNPC. (**A**) Western blot showing the expression of DDX5, DDX17 and HNRNPC following the treatment with siRNA targeted against luciferase (negative control), DDX5/DDX17 and HNRNPC. Quantification of this experiment is shown in Supplementary Figure S7A. (**B**) RT-PCR analysis showing the inclusion of a selection of alternative exons after the depletion of DDX5/DDX17 and/or HNRNPC in 293T cells. The corresponding gene and exon number (according to FasterDB annotation) are indicated. Exons down-regulated and up-regulated upon SF knockdown are shown in the left and right column, respectively. (**C**) Quantification of the RT-PCR experiment. The indicated ΔPSI values correspond to the difference between the PSI value of each depleted sample and the control sample. The statistical comparison between each condition (including the unshown control condition) was calculated using a one-way analysis of variance (ANOVA) (Holm–Sidak’s multiple comparison tests: *P < 0.05, **P < 0.01, ***P < 0.001). (**D**) Co-immunoprecipitation assays between endogenous HNRNPC and DDX17 in 293T cells, in the absence of presence of RNase A.

Finally, to experimentally validate our predictions, we silenced the expression of DDX5/DDX17 and HNRNPC in 293T cells, independently or together (Figure 4A and Supplementary Figure S7A), and we monitored the effect of these treatments on a selection of alternative exons whose inclusion was found to be impacted by the knockdown of these factors (10 more skipped exons and 7 more included exons). In conditions of single depletion, the inclusion of all exons was modified according to the predictions, and interestingly, the combined depletion of DDX5/DDX17 and HNRNPC significantly enhanced the effect of single siRNA treatment on splicing (Figures 4B and C). We observed the same cooperative effect of DDX5/DDX17 and HNRNPC on exon inclusion in the neuroblastoma SH-SY5Y cell line (Supplementary Figures S7B–D). This cooperation prompted us to test whether these factors interact with each other. Indeed, endogenous HNRNPC and DDX17 co-immunoprecipitated in an RNA-independent manner in 293T cells, suggesting a possible direct interaction (Figure 4D). No clear sign of interaction was observed between DDX5 and HNRNPC (Supplementary Figure S7E).

**Alt text for **  Figure 4: Experiments validating the cooperative effect of DDX5/DDX17 helicases and HNRNPC on alternative exon inclusion and the interaction between DDX17 and HNRNPC in HEK293 cells.

Altogether, these results disclosed a novel functional partnership between HNRNPC and the helicase DDX17 and illustrated the usefulness of SplicingLore to improve our knowledge on splicing regulation.

## Discussion

One challenge faced by scientists from the alternative splicing field is to decode the cooperative or antagonistic effects of SFs to understand and eventually predict splicing outcomes on a genome-wide scale (22). Exploiting a large collection of RNA-seq-derived lists of exons regulated by 75 SFs, SplicingLore helps to fill this knowledge gap in a straightforward manner. RNA-seq datasets from ENCODE have previously been analysed in a transverse manner, for example along with RBP-RNA interactions datasets, which helped to understand the functional roles of these proteins in alternative splicing (11, 12). SplicingLore includes not only 100 ENCODE datasets but also 60 datasets from other sources. Our resource also enables cross-comparisons between datasets and allows to directly search for SFs that control the inclusion of a list of exons provided by the user. Therefore, SplicingLore complements other splicing databases, such as VastDB (5) or ASpedia (6), that are repositories of alternative splicing profiles and inclusion levels across cell types or organisms, which also provide useful information about the functional impact of splicing variations. SplicingLore only requires to format correctly the list of exons of interest, and it is therefore fast and easy to run. All datasets were selected based on the quality and coverage depth of the sequencing experiment, and they were processed in a standardized manner. This unique experimental workflow ensures a reliable calculation of correlation scores between the tested dataset and good validation efficiency when testing the inclusion of the predicted exons experimentally.

Of note, what we describe here is the most straightforward way to use SplicingLore, but our data can be exploited in a different and deeper way, depending on the user’s interest. For example, we previously used some of the SF-specific alternative exon lists stored in SplicingLore to explore the intrinsic nature of these exons and the links that exist between their biased nucleotidic composition and splicing regulatory sequences (23, 24).

As a proof of concept, we searched for SFs that could stimulate or antagonize the inclusion of alternative exons regulated by RNA helicases DDX17 and DDX5. These closely related paralog proteins belong to the large family of evolutionarily conserved DEAD-box ATP-dependent RNA helicases (25). Previous reports, from our lab and others, have shown that DDX5 and DDX17 control the inclusion of a large number of exons by modulating the folding of their target transcripts, thanks to their helicase activity, and by modulating the binding of splicing regulators to RNA (21, 26–31). This is the case of HNRNPH for example, whose binding to RNA is facilitated by DDX5/DDX17 (21), and which was predicted by SplicingLore among the top predicted SFs for positive correlation with the helicases.

SplicingLore predicted a good correlation in the regulation of several hundreds of exons by DDX5/DDX17 and HNRNPC, including exons that are either activated or repressed by those factors. We experimentally validated the additive effect of these factors on a subset of both classes of exons, in two different cell lines, and found that HNRNPC and DDX17 associate with each other in cells in an RNA-independent manner. The molecular nature of this functional relationship is not clear, and understanding how HNRNPC stimulates exon inclusion while it is often described as a splicing repressor (12) will require further investigation.

## Conclusion

SplicingLore represents a straightforward and reliable tool to investigate alternative splicing regulation by a large panel of SFs. SplicingLore also complements other web resources developed by our group, FasterDB (7) and Exon Ontology (8), which together form a suite of tools to facilitate the understanding of alternative splicing, from its regulation to its biological consequences. SplicingLore will be useful not only to splicing specialists but also to scientists less familiar with the complex mechanisms that underlie splicing regulation.

## Materials and Methods

### Database and web interface

SplicingLore database was implemented on Linux Ubuntu 22.04, using a MySQL database (www.mysql.com) and organized in nine tables. The web interface was developed using the programming languages PHP, CSS and JavaScript. Tools performing statistical tests were driven in R (v4.2.0) using R packages Tidyverse (doi.org/10.21105/joss.01686) or in Python (v3.10) using Plotly (32) for graphics.

### Data collection

SplicingLore is a database of 160 public datasets retrieved from GEO omnibus (16) and ENCODE consortium (17), linked to a user-friendly web interface (Supplementary Table S1). All datasets were derived from Illumina RNA-seq experiments performed in 21 different human cell lines (293T, 786-O, A498, A673, Endoc-BH1, GM19238, HeLa, HepG2, SH-SY5Y, HMLE, Huh-7, K562, MCF7, MDA-MB-231, MG63, RD, RH30, RH41, RKO, LNCaP, and LM2) in which the expression of 75 SFs was inhibited by siRNA or shRNA treatment. The quality control of the sequencing data was evaluated using FastQC (v0.11.9). Reads were trimmed using Prinseq-lite (v0.20.4) (33) (‐‐trim‐right 20) and filtered by average quality score (‐‐trim‐qual 20) and cutadapt (v1.18) (34). Sequencing projects for which the filtered read count was lower than 20 millions pairs of reads were eliminated.

### Differential exon inclusion analysis

For each dataset, we performed a qualitative and quantitative analysis of splicing variations using the FaRLine tool (7) (hg19 genome), comparing the condition in which the expression of the SF was inhibited to the control condition. The following parameters were used to define exons that were differentially included: |ΔPSI| ≥ 10%, adjusted *P*-value ≤ 0.05. The ΔPSI corresponds to the change in exon inclusion between two conditions. We then retrieved and fed the SplicingLore database with the lists of differentially included cassette exons corresponding to each dataset.

### Clustering analyses

Heatmaps of Supplementary Figures S1 and S2 were created with R (v4.2.0) using R packages Tidyverse (doi.org/10.21105/joss.01686) and pheatmap (https://github.com/raivokolde/pheatmap).

### Randomization test and scoring

- Input list of exons with ΔPSI and *P*-values

We defined a Pearson correlation between the ΔPSI of two sets of exons. The computed empirical *P*-value is the probability of observing a Pearson correlation as high or higher as the observed correlation when considering a set of randomly associated exons. This probability was computed from the empirical cumulative distribution function generated by computing the Pearson correlation for 10^4^ random sets of exon (which leads to a maximal *P*-value resolution of 10^−4^).

The ‘percent.sig.input’ is the fraction of significantly regulated exons from the input list of exons that are also found in the list of exons regulated by the indicated SF. The ‘percent.sig.SF’ is the fraction of significantly regulated exons from the list of exons regulated by the indicated SF that are also found in the input list of exons.

A confidence score (Score) was set up to facilitate the identification of candidate SF. It is linked to a permutation statistic test, which also gives a *P*-value, the ‘percent.sig.input’ and the ‘percent.sig.SF’. The Score ranges between 0 and 1, which indicates the degree of correlation or anti-correlation between the effect of a given SF and the input list of exons provided by the user.


$${{Score}}\!=\!{{mean}}\left( {1 - p{{value}},\frac{{{{percent}}{{.sig}}{{.input}}}}{{100}},\frac{{{{percent}}{{.sig}}{{.sf}}}}{{100}}} \right).$$

- Basic input list of exons (chromosome coordinates only)

For an input set of exons *E*, randomization tests were performed to test if the number of exons *N* regulated by a given SF *S* is enriched or impoverished. For this, 10^4^ sets of control exons with the same size as *E* were sampled. Then the number of exons regulated by *S* was computed for each control set. Finally, an empirical *P*-value was computed for *S* as:


$${P_{{{emp}}}} = \frac{{{{min}}\left( {k,l} \right) + 1}}{{10,000 + 1}},$$

where *k* is the number of controls sets with a number of exons regulated by *S* higher or equal to *N*, and *l* is the number of controls sets with a number of exon regulated by *S* lower or equal to *N*. This *P*-value was computed for each SF and was then corrected using the Benjamini–Hochberg procedure.

### Analysis of CLIP-seq data

Processed bed peak files from various CLIP-seq experiment projects, targeting 29 different SFs in different cell lines, were recovered from various sources, mainly POSTAR2 (35) and ENCODE (36), see Supplementary Table S8. Peaks obtained from experiments targeting the same SF were merged together using BEDtools (37). We then defined the sets of exons in the Splicing Lore database that were significantly more included or skipped by the 29 SFs that were knocked down in at least one sample. Exons that were significantly regulated in opposite ways by the same SF in different samples were removed. Factors with less than 100 significantly more included and skipped exons were discarded. The ratio *R_i_* was then calculated for a SF *i*:


$${R_i} = {\mathrm{log}}2\left( {\frac{{{P_{reg}} + 0.01}}{{{P_{ctrl}} + 0.01}}} \right)$$

where *P*_reg_ is the proportion of exons more included or skipped upon knockdown of a given SF that overlap a peak of that SF and *P*_ctrl_ is the proportion of control exons that overlap the same peaks. Control exons correspond to all other exons detected by FaRLine in at least one project, excluding those significantly skipped or included upon knockdown of factor *i*. Finally, logistic regression was performed to test whether the proportions *P*_reg_ and *P*_ctrl_ were significantly different. All calculated *P-*values were corrected using the Benjamini–Hochberg procedure (38). Non-significant *R_i_* are shown in grey. The same method was applied to six 100-nucleotide regions surrounding regulated exons.

### Analysis of RNA binding motifs

The binding motifs preferentially recognized by 21 human SF were recovered from mCrossBase (https://zhanglab.c2b2.columbia.edu/mCrossBase/) (39) and cisBP-RNA Database (http://cisbp-rna.ccbr.utoronto.ca/) (40). Supplementary Table S9 describes the binding motifs used for further analysis and converted to MEME format. The set of sequences corresponding to significantly more included or skipped exons upon knockdown of each of these 21 SF was recovered. In addition, for each of these sets, a control set of exon sequences was defined, corresponding to all other exons detected by FaRLine in at least one project, excluding those that were significantly regulated upon knockdown of the SF. These sequences were then extended by 200 nucleotides in both directions. A motif enrichment analysis was performed with the simple enrichment analysis (SEA) program (41) using the binding motif, the extended sequences regulated by a given SF and control sequences. As a negative enrichment is not calculated by the SEA tool, motif impoverishment analysis was performed using the same principle but with the regulated sequences as control and control sequences as primary sequences. The *V* value was then calculated for each set of exons up- or down-regulated upon knockdown of an SF, using the following formula:


$$V = {{min}}\left( {1 - {P_e},1 - {P_i}} \right) \times s{{With}}\{ \begin{array}{*{20}{c}}
{s = - 1}&{{\mathrm{if}}}&{{P_i} \lt {P_e}}\\
{s = 1}&{{\mathrm{if}}}&{{P_i} \gt {P_e}}
\end{array},$$

where *P_e_* is the SEA *P-*value for positive enrichment of a binding motif in regulated sequences by a given SF and *P_i_* is the same type of *P-*value but for negative enrichment. Finally, a heatmap was generated showing these *V*-values for exons that were up- or down-regulated in each SF knockdown. *V*-values above and below 0.95 are shown in red and blue, respectively. Non-significant *V*-values (between −0.95 and 0.95) are shown in grey, and *V*-values for SF with less than 100 exons regulated upon knockdown are shown in white.

### Cell culture and transfections

Human embryonic kidney 293T and SH-SY5Y cells were grown as recommended by the manufacturer and transfected as described previously (26, 42). For knockdown experiments, we used a total of 40 nM siRNA as follows: 40 nM siCtrl for control experiments, 20 nM of si*DDX5/DDX17* or si*HNRNPC* + 20 nM siCtrl for single factor depletion or 20 nM si*DDX5/DDX17* + 20 nM si*HNRNPC* for double knockdown. Cells were harvested 48 h later. Sequences of siRNAs are given in Supplementary Table S2.

### Co-immunoprecipitation and Western blotting

Total protein extraction was carried out as previously described (21). Primary antibodies used for Western blotting: DDX5 (ab10261, Abcam), DDX17 (ab24601, Abcam), HNRNPC (D6S3N, Cell Signaling) and GAPDH (sc-1616, SantaCruz).

For co-immunoprecipitation, cells were harvested and gently lysed for 5 min on ice in a buffer containing 10 mM Tris-HCl pH 8.0, 140 mM NaCl, 1.5 mM MgCL_2_, 10 mM EDTA and 0.5% NP40, completed with protease and phosphatase inhibitors (Roche #11697498001 and #5892970001), to isolate the nuclei from the cytoplasm. After centrifugation, the nuclei were lysed in the IP buffer (20 mM Tris-HCl pH 7.5, 150 mM NaCl, 2 mM EDTA, 1% NP40 and 10% glycerol and protease/phosphatase inhibitors) for 30 min at 4°C under constant mixing. The nuclear lysate was centrifuged for 15 min to remove debris, and soluble proteins were quantified by BCA (Thermo Fisher Scientific). The lysate was pre-cleared with 30 μL of Dynabeads Protein G (Thermo Fisher Scientific) for 30 min under rotatory mixing and then split in 1.5 mg aliquots of proteins for each assay. Each fraction received 5 µg of antibody, and the incubation was left overnight at 4°C under rotation. The following antibodies were used for IP: rabbit anti-DDX17 (19910-1-AP, ProteinTech) and anti-HNRNPC (PA522280, Thermo Fisher Scientific) or a control rabbit IgG (Thermo Fisher Scientific), goat anti-DDX5 (ab10261, Abcam) or control goat IgG (Santa Cruz). The next day, the different lysate/antibody mixtures were incubated with 50 µl Dynabeads Protein G (Thermo Fisher Scientific) blocked with BSA, for 4 h at 4°C under rotation. Beads were then washed five times with IP buffer. Elution was performed by boiling for 5 min in SDS-PAGE loading buffer prior to analysis by Western blotting.

### RNA extraction and PCR analyses

Total RNA was isolated using TriPure Isolation Reagent (Roche). For reverse transcription, 2 µg of purified RNAs were treated with Dnase I (Thermo Fisher Scientific) and retrotranscribed using Maxima reverse transcriptase (Thermo Fisher Scientific), as recommended by the manufacturer. Potential genomic DNA contamination was systematically verified by performing negative RT controls in absence of enzyme and by including controls with water instead of cDNA in PCR assays. All PCR analyses were performed on 0.5 ng cDNA using 0.5 U GoTaq® DNA polymerase (Promega). Quantification of PCR products was performed using the Image Lab software (BioRad) after agarose gel electrophoresis. The PSI value of each alternative exon was calculated in each condition using the following formula: inclusion product/(inclusion product + skipping product) × 100. The ΔPSI corresponds to the difference between the PSI for each silencing condition and the PSI of the siCtrl condition. Sequences of all primers are given in Supplementary Table S2.

## Supplementary Material

baad091_Supp

## Data Availability

All raw data supporting the findings of this study (Supplementary Table 1) were retrieved from GEO omnibus^16^ and ENCODE consortium^17^. All processed data can be freely retrieved from the SplingLore database.

## Code availability

The code used to process the data presented in the manuscript is available here: https://github.com/helenepolveche/splicinglore_website.
