# Supplementary material for: SplicingLore: a web resource for studying the regulation of cassette exons by human splicing factors
Source: Database (Oxford). 2023 Dec 21;2023:baad091. doi: 10.1093/database/baad091 (PMC10735282; doi:10.1093/database/baad091)
Supplement: baad091_Supp [file baad091_supp.zip › suppl_data/revised_figures_sup.pptx]

## Slide 1
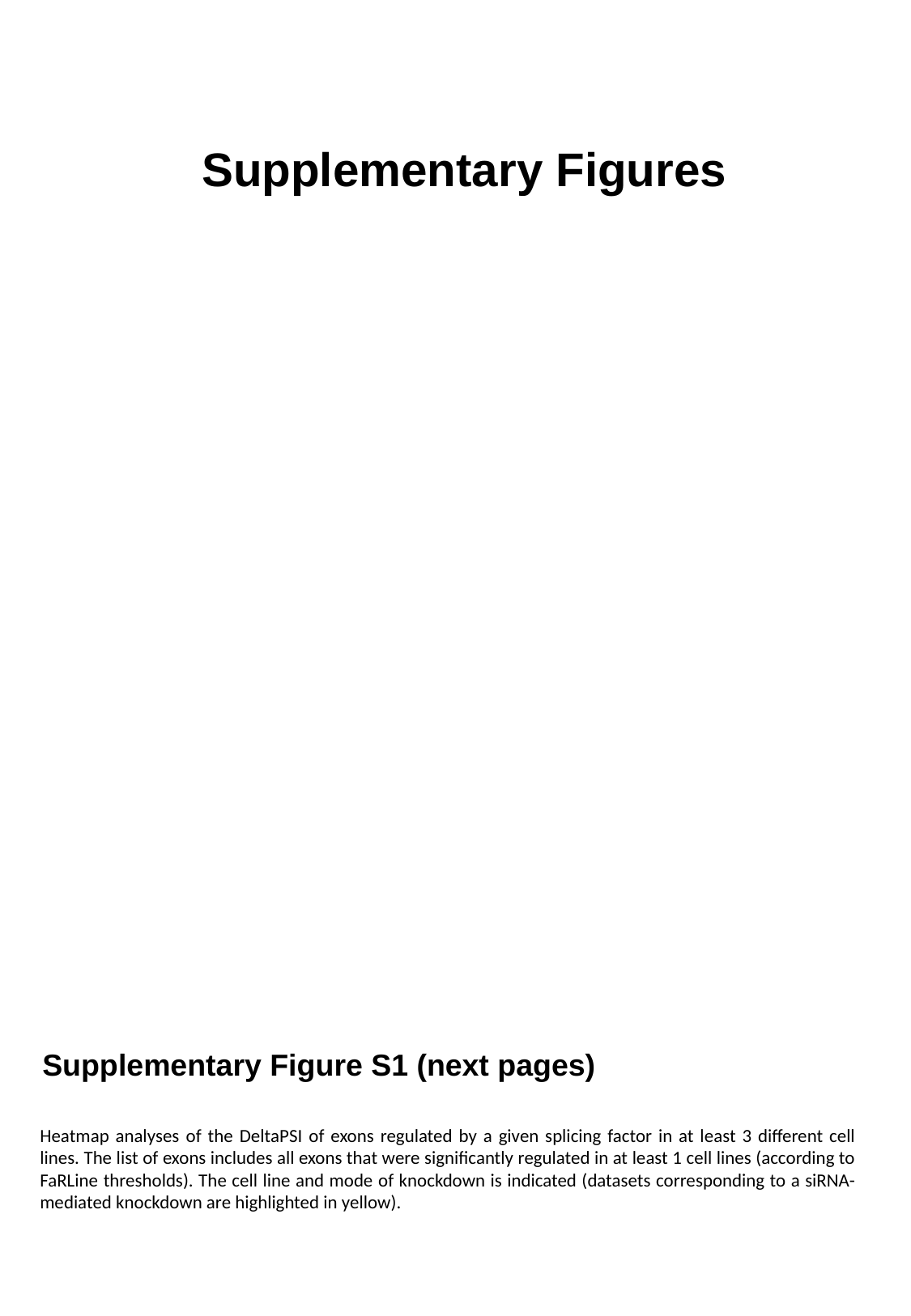

Supplementary Figures
Supplementary Figure S1 (next pages)
Heatmap analyses of the DeltaPSI of exons regulated by a given splicing factor in at least 3 different cell lines. The list of exons includes all exons that were significantly regulated in at least 1 cell lines (according to FaRLine thresholds). The cell line and mode of knockdown is indicated (datasets corresponding to a siRNA-mediated knockdown are highlighted in yellow).

## Slide 2
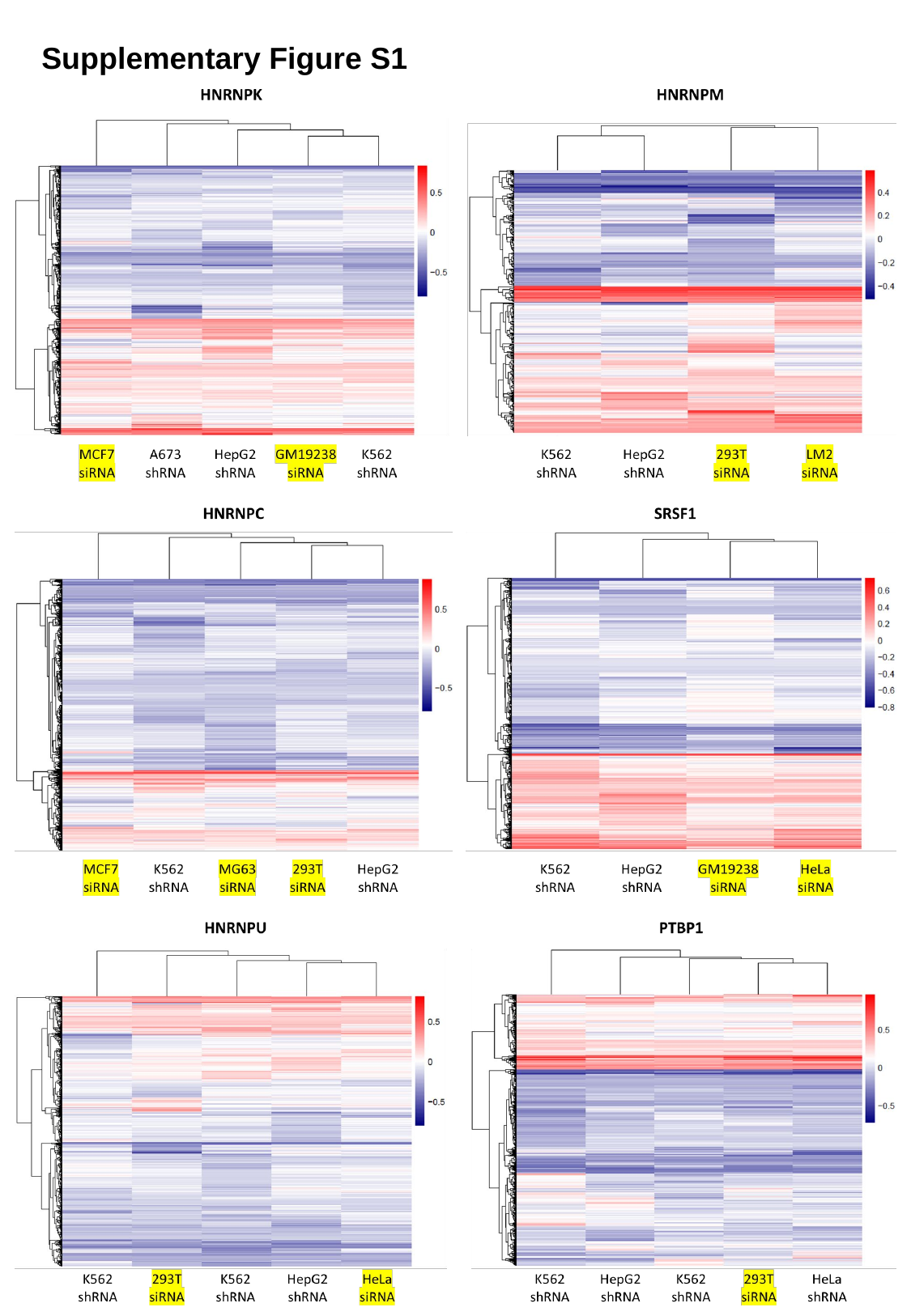

Supplementary Figure S1

## Slide 3
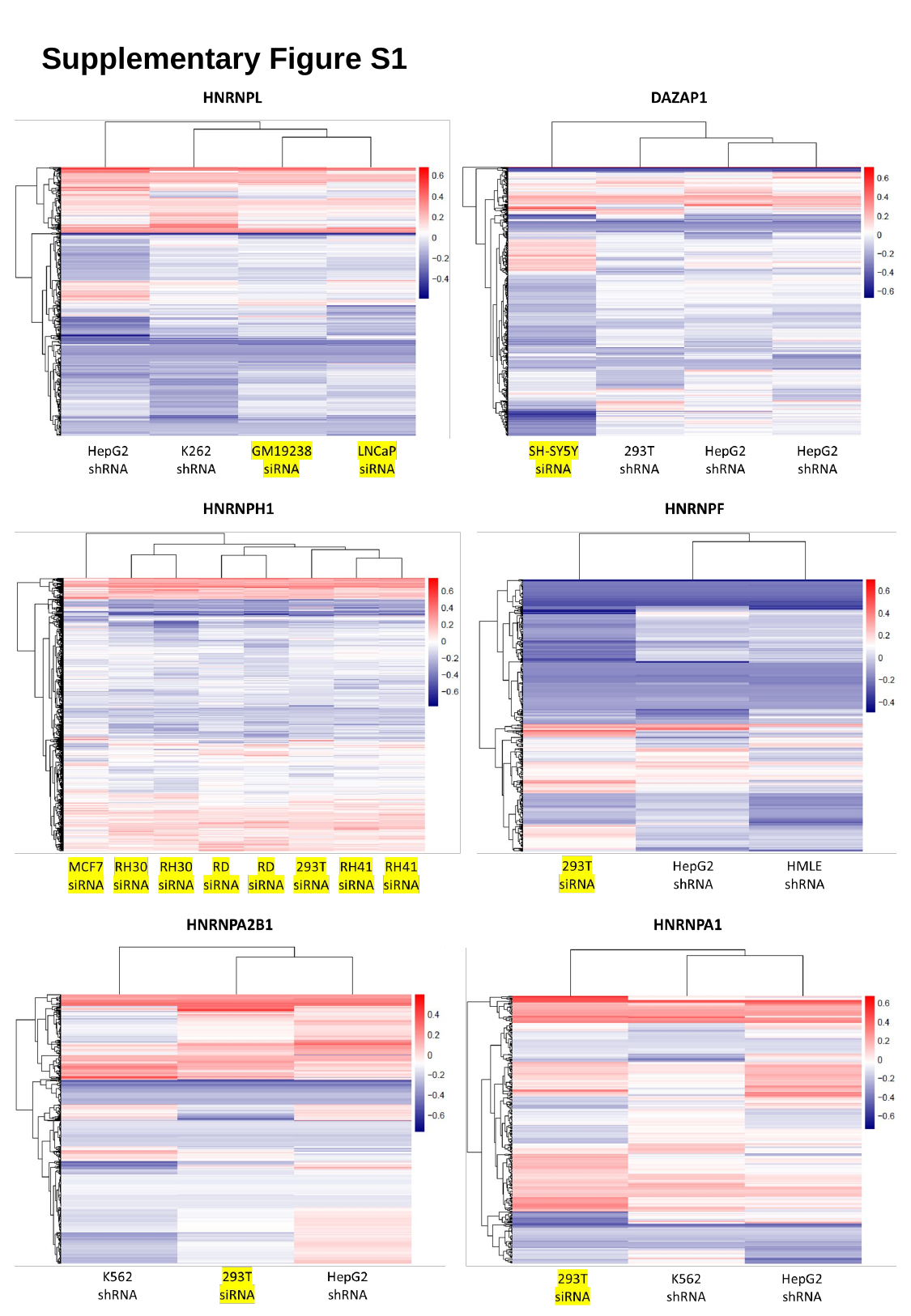

Supplementary Figure S1

## Slide 4
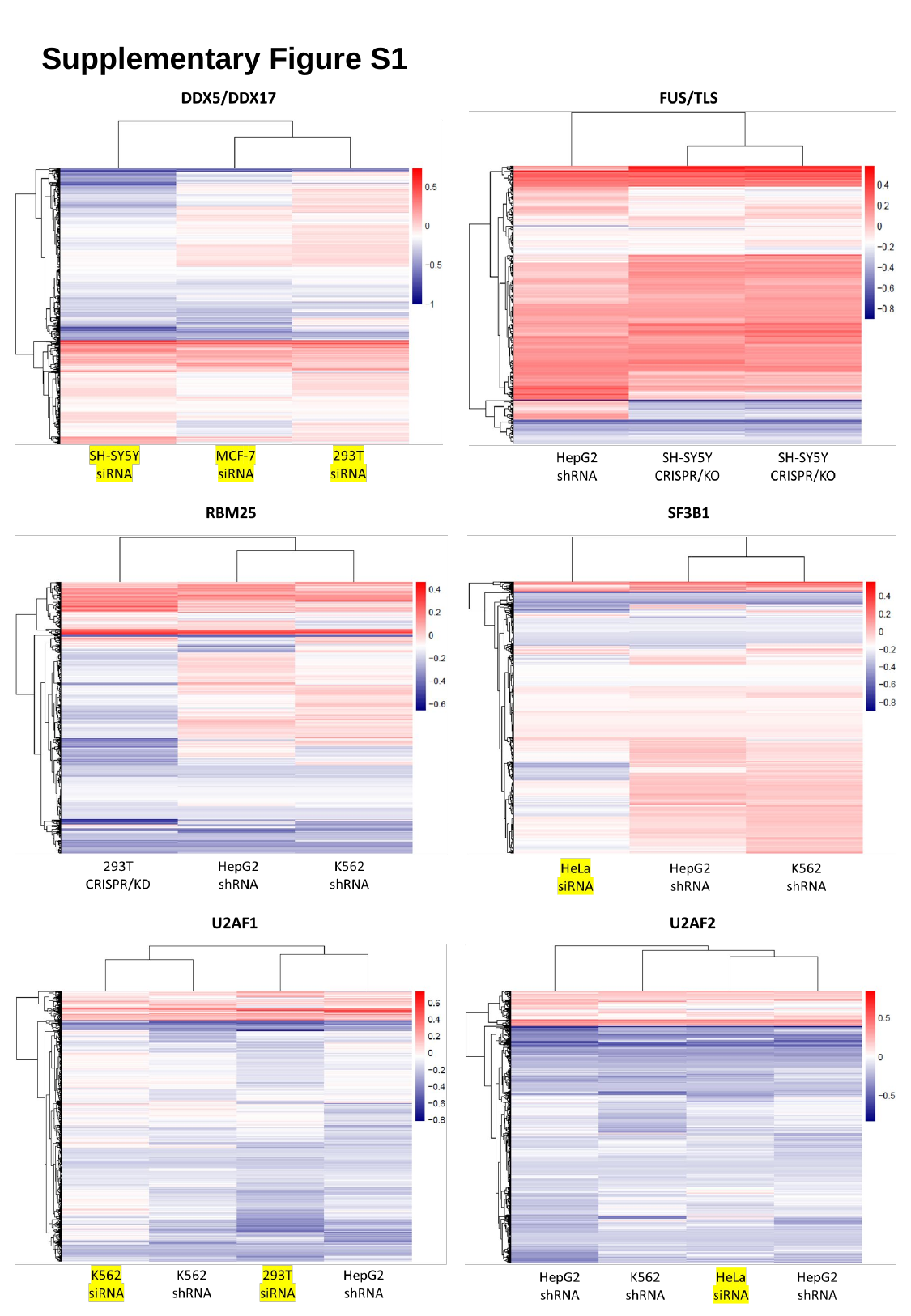

Supplementary Figure S1

## Slide 5
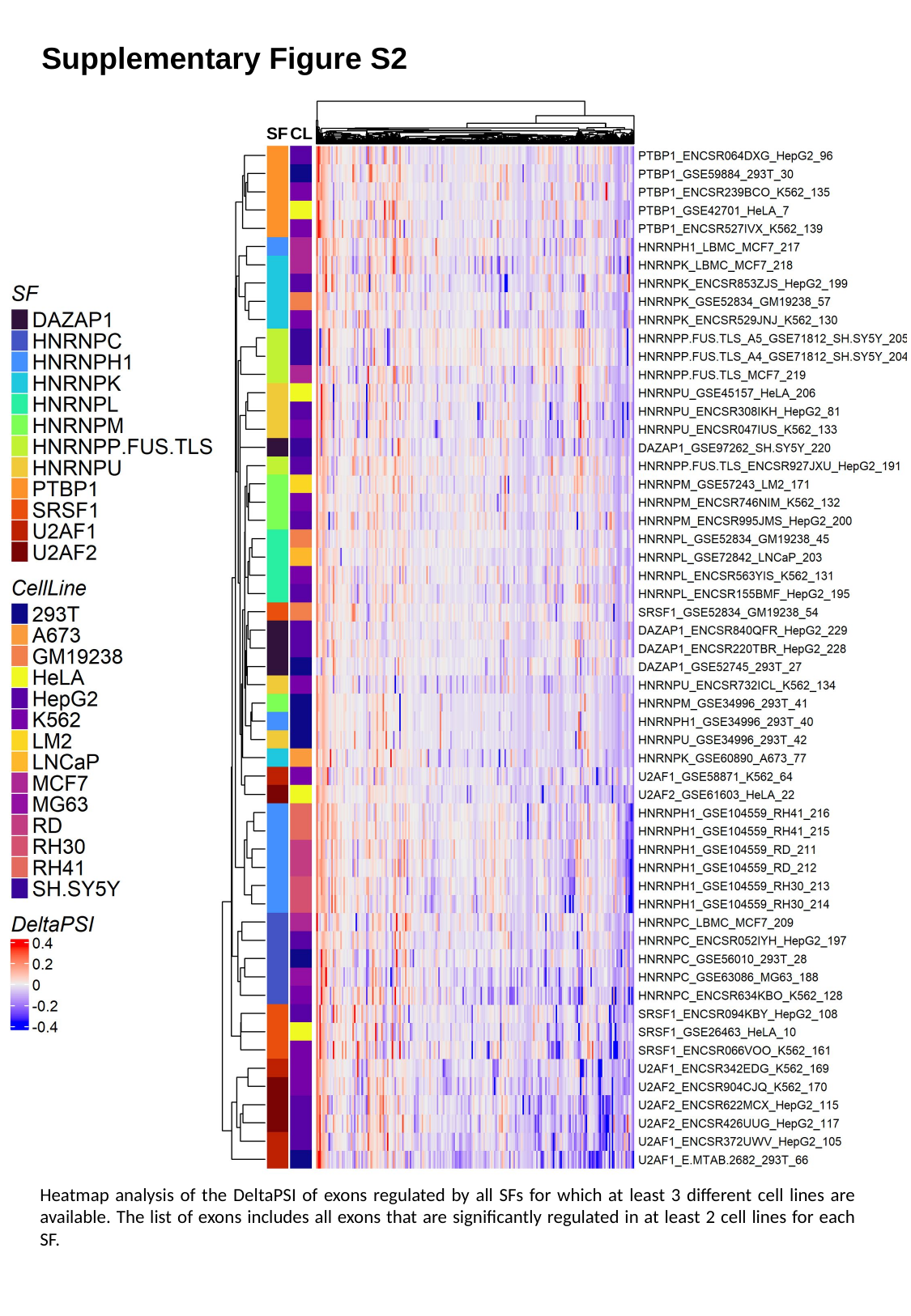

Supplementary Figure S2
SF
CL
Heatmap analysis of the DeltaPSI of exons regulated by all SFs for which at least 3 different cell lines are available. The list of exons includes all exons that are significantly regulated in at least 2 cell lines for each SF.

## Slide 6
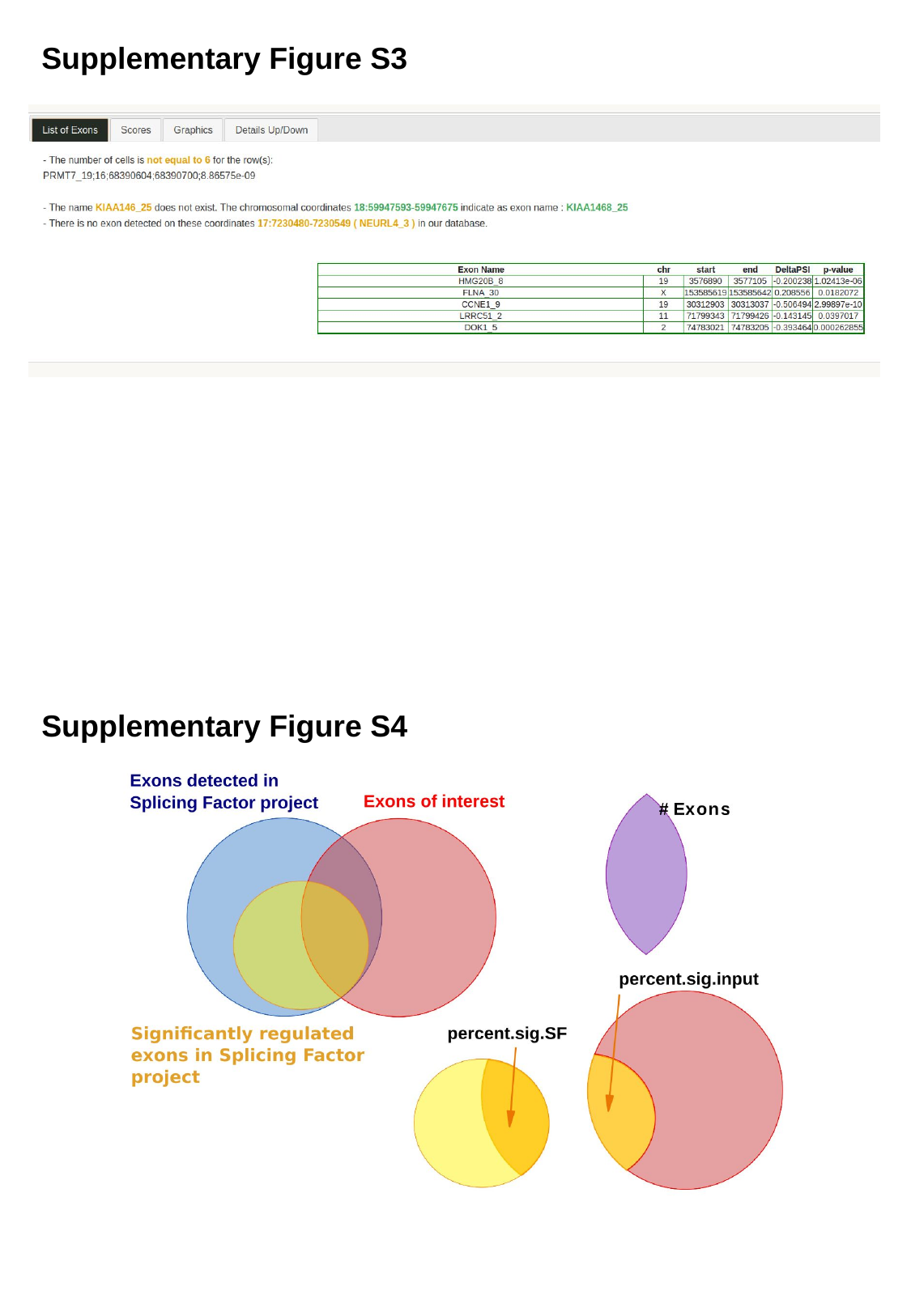

Supplementary Figure S3
Supplementary Figure S4

## Slide 7
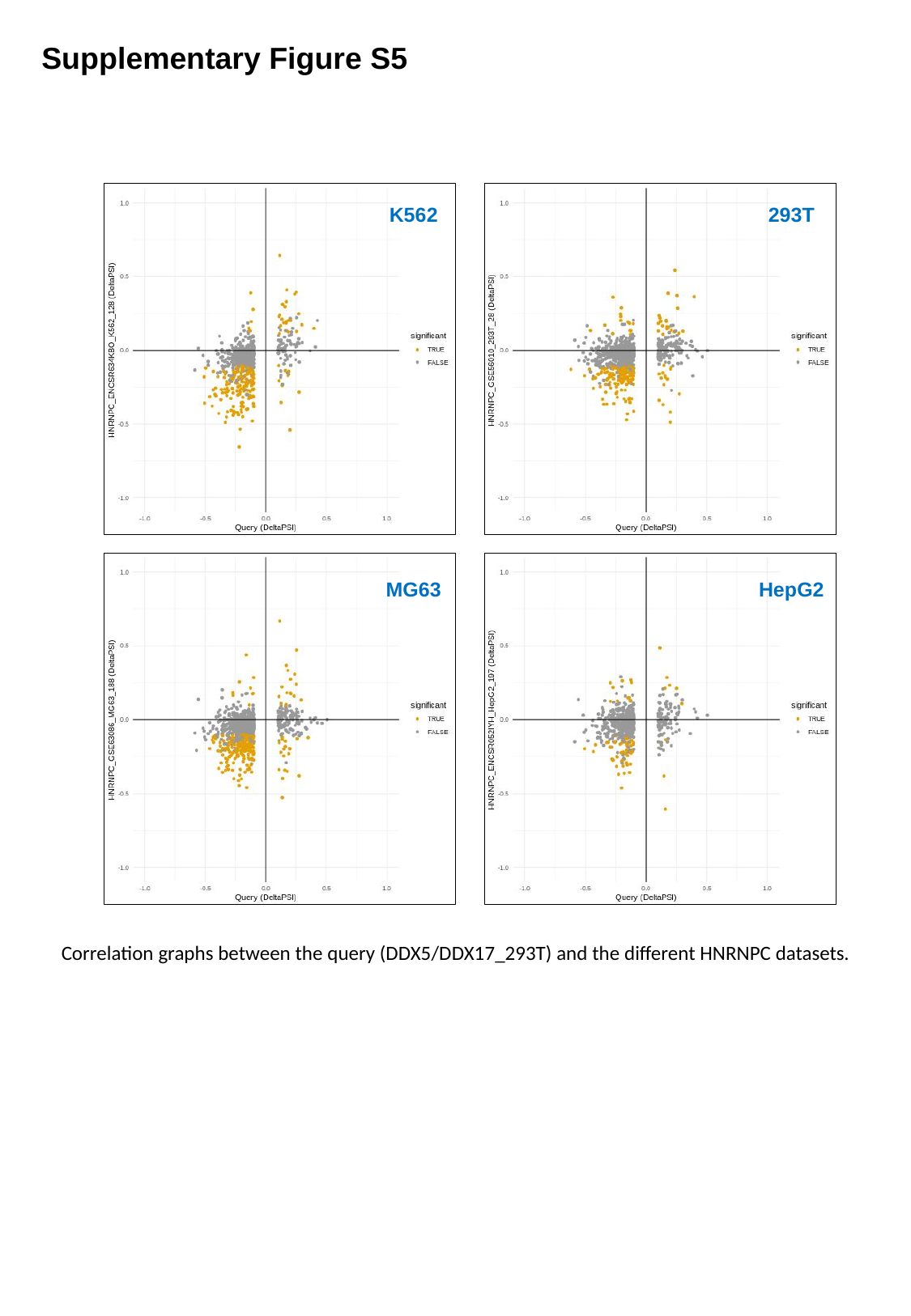

Supplementary Figure S5
K562
293T
MG63
HepG2
Correlation graphs between the query (DDX5/DDX17_293T) and the different HNRNPC datasets.

## Slide 8
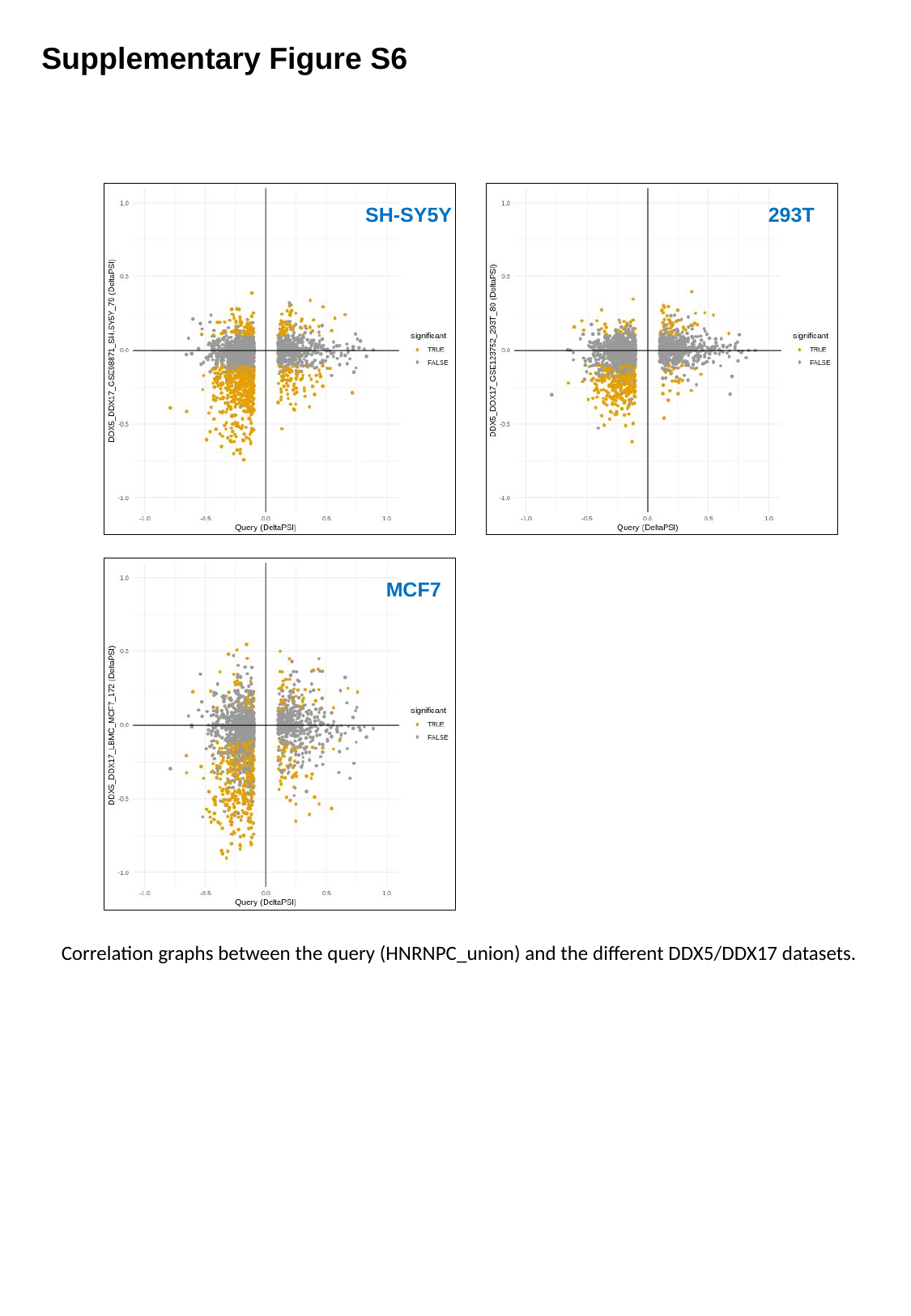

Supplementary Figure S6
SH-SY5Y
293T
MCF7
Correlation graphs between the query (HNRNPC_union) and the different DDX5/DDX17 datasets.

## Slide 9
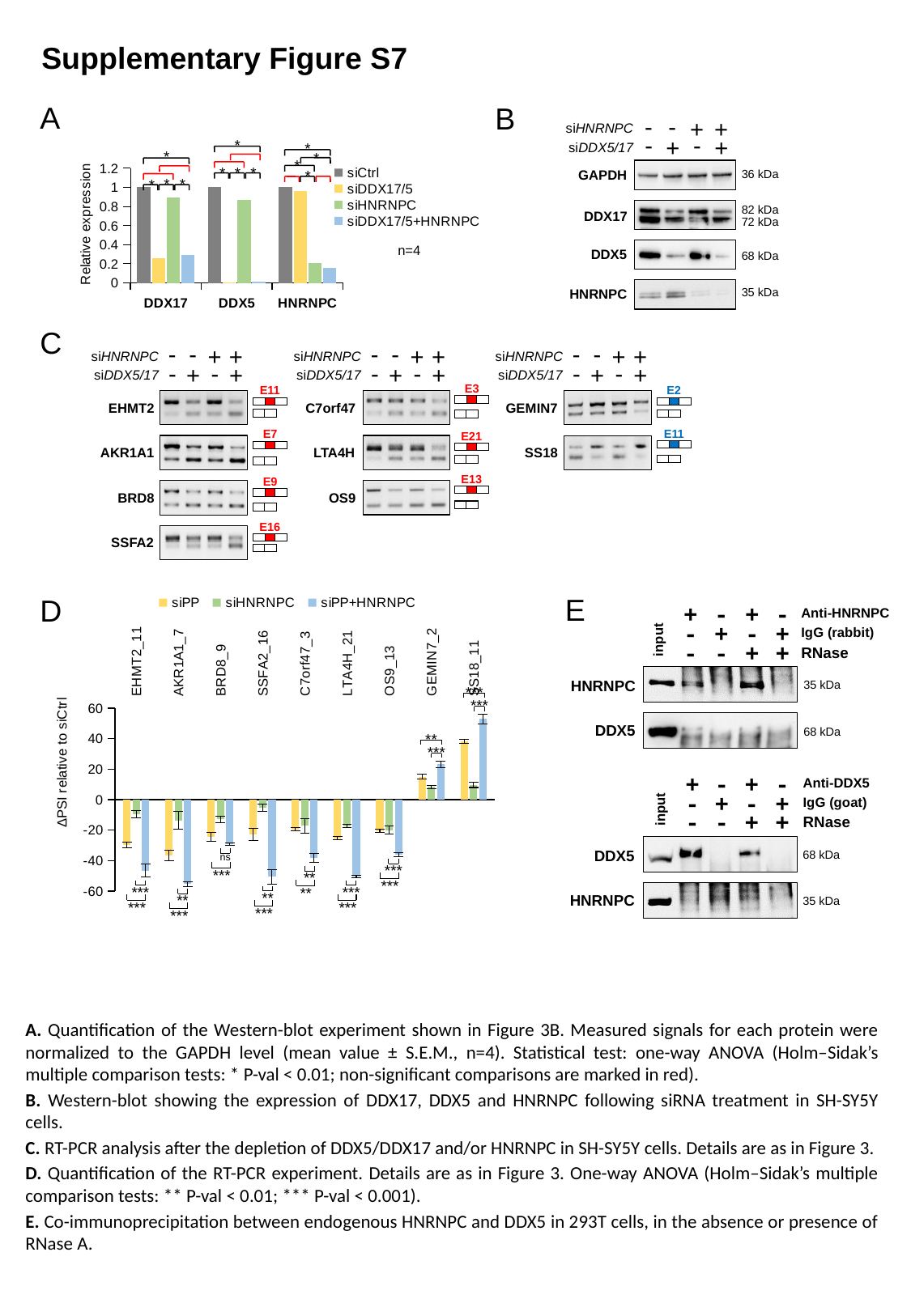

Supplementary Figure S7
A
B
-
-
+
+
siHNRNPC
-
-
+
+
siDDX5/17
36 kDa
GAPDH
82 kDa
DDX17
72 kDa
DDX5
68 kDa
35 kDa
HNRNPC
*
*
*
*
### Chart
| Category | siCtrl | siDDX17/5 | siHNRNPC | siDDX17/5+HNRNPC |
|---|---|---|---|---|
| DDX17 | 1.0 | 0.25642388225324 | 0.896077238573007 | 0.289871363350848 |
| DDX5 | 1.0 | 0.00820020396607368 | 0.872096426611116 | 0.00952921511149329 |
| HNRNPC | 1.0 | 0.964540317903078 | 0.21127654069507 | 0.158529786246815 |
*
*
*
*
*
*
*
*
n=4
C
-
-
+
+
siHNRNPC
-
-
+
+
siDDX5/17
E11
EHMT2
E7
AKR1A1
E9
BRD8
E16
SSFA2
-
-
+
+
siHNRNPC
-
-
+
+
siDDX5/17
E3
C7orf47
E21
LTA4H
E13
OS9
-
-
+
+
siHNRNPC
-
-
+
+
siDDX5/17
E2
GEMIN7
E11
SS18
E
D
### Chart
| Category | siPP | siHNRNPC | siPP+HNRNPC |
|---|---|---|---|
| EHMT2_11 | -29.79598969074841 | -9.578078080886058 | -46.64169748785921 |
| AKR1A1_7 | -36.439853563059486 | -13.665669568273685 | -55.38750282673842 |
| BRD8_9 | -24.413350028978783 | -12.998702125987755 | -29.210962513793504 |
| SSFA2_16 | -22.846134994238287 | -5.458419708841343 | -50.57891627561927 |
| C7orf47_3 | -19.467912071333192 | -17.145066296675044 | -38.35089909666286 |
| LTA4H_21 | -25.408423254508012 | -17.336114845734542 | -50.35243223921197 |
| OS9_13 | -20.51607618464301 | -19.94865821428641 | -35.964946850341086 |
| GEMIN7_2 | 15.05249862859715 | 8.35083245459576 | 23.09203041596011 |
| SS18_11 | 38.065639781822235 | 9.625480451064552 | 52.887115904687086 |***
***
**
***
ns
***
***
**
***
***
***
**
**
**
***
***
***
***
+
-
+
-
Anti-HNRNPC
-
+
-
+
IgG (rabbit)
input
-
-
+
+
RNase
HNRNPC
35 kDa
DDX5
68 kDa
+
-
+
-
Anti-DDX5
-
+
-
+
IgG (goat)
input
-
-
+
+
RNase
DDX5
68 kDa
HNRNPC
35 kDa
A. Quantification of the Western-blot experiment shown in Figure 3B. Measured signals for each protein were normalized to the GAPDH level (mean value ± S.E.M., n=4). Statistical test: one-way ANOVA (Holm–Sidak’s multiple comparison tests: * P-val < 0.01; non-significant comparisons are marked in red).
B. Western-blot showing the expression of DDX17, DDX5 and HNRNPC following siRNA treatment in SH-SY5Y cells.
C. RT-PCR analysis after the depletion of DDX5/DDX17 and/or HNRNPC in SH-SY5Y cells. Details are as in Figure 3.
D. Quantification of the RT-PCR experiment. Details are as in Figure 3. One-way ANOVA (Holm–Sidak’s multiple comparison tests: ** P-val < 0.01; *** P-val < 0.001).
E. Co-immunoprecipitation between endogenous HNRNPC and DDX5 in 293T cells, in the absence or presence of RNase A.
